# The relationship between psychophysical body categorization performance and male body dissatisfaction

**DOI:** 10.1038/s41598-019-40502-z

**Published:** 2019-03-07

**Authors:** Daniel Talbot, Evelyn Smith, John Cass

**Affiliations:** School of Social Sciences and Psychology, Western Sydney University, Bullecourt Ave, Milperra, 2214 Australia

## Abstract

The present study compared the predictive relationship between various psychophysical indices of body categorization performance (*Point of Subjective Equivalence* (PSE), *Just Noticeable Difference* (JND) and *Reaction Time* (RT)) and male body dissatisfaction (*Male Body Attitudes Scale* (MBAS)) and eating disorder symptoms (*Eating disorders examination questionnaire* (EDE-Q)), with performance on a validated figure rating scale (*Visual Body Scale for Men* (VBSM)). Body Mass Index, body fat percentage, and fat free mass index were also measured. PSE was not as sensitive in predicting body dissatisfaction and eating disorder symptoms as the VBSM. JND and average RT were found to be sensitive predictors of body dissatisfaction and eating disorder symptoms across the 82 male participants. JND proved to be a better indicator of weight concern than the VBSM-M. Whilst the body categorization task offers new insights into the way body images may be processed by males with different levels of body dissatisfaction, the VBSM and the conventional self-report measures are likely to be clinically more efficacious at measuring body dissatisfaction.

## Introduction

Body dissatisfaction is defined as negative evaluation of one’s body size and/or shape. This definition can also be extended to specific facets of body image such as muscle size, muscle tone, weight, and percentage of body fat^1^. In recent years there has been an increased focus on male body dissatisfaction due to its clinical association with eating disorder symptoms^2,3^, obesity^4,5^, and muscle dysmorphia^6^. These associations are of particular concern when considering the high incidence of reported body dissatisfaction amongst males^7,8^. Frederick, *et al*.^9^ reported that up to 71% of undergraduate males are dissatisfied with their level of body fat and 90% want to increase their muscularity.

A common tool for measuring body dissatisfaction is the figural rating scale^1^. When employed clinically, figural rating scales utilize a simultaneously presented set of human male body images graded from small (far left) to large (far right) in terms of body fat and/or muscular bulk. Participants indicate which body best represents their *perceived* body, and which best represents their *desired* body. The discrepancy between a given participant’s selected *perceived* body and their *desired* body is used as an index of body dissatisfaction, with larger scores indicating greater body dissatisfaction^1^. Figural rating scales are advantageous as they allow for an unambiguous visual selection of body image, as opposed to written choice descriptors often employed in self-report questionnaires. For example, consider the following item from the Eating Disorder Examination Questionnaire (EDE-Q)^10^: “*Have you had a definite desire to have a totally flat stomach?*”. When responding to this item, it is ambiguous to which biometric factor (i.e. body fat or muscle) the item refers. Thus, this question could be interpreted in a variety of ways, resulting in inconsistent responses between individuals. Additionally, figural rating scales help to control for varying literary and/or language ability between participants. That said, existing figural rating scales hold several limitations. Gardner, Friedman, and Jackson^11^ observed that the manner in which figural rating scales are presented inflates test-retest reliability. As these scales typically present the various body images simultaneously and in ascending order (e.g. thin body shapes on the left, obese body shapes on the right), participants may plausibly use their initial selection’s spatial location within this scale to inform their subsequent judgments. Further criticisms of existing figural rating scales include: lack of resolution within a given body dimension (with most scales presenting fewer than 10 body images)^11,12^; too few trial presentations – often just single-trials; poor quality and realism of body images; a lack of muscularity variation; and inclusion of ethnicity-specific facial features^12^. Additionally, figural rating scales hold disadvantages common to all self-report measures such as response biases (e.g. social desirability bias).

In order to address some of these limitations and to investigate whether we can achieve a more objective measure of body dissatisfaction, the present study seeks to utilize a variation of Fechner’s method of constant stimuli (MOCS)^13^ – a classical psychophysical technique that overcomes some of methodological shortcomings of figural rating scales mentioned above. In a typical MOCS task the participant is presented with a sequence of experimental trials, each depicting a stimulus which varies randomly along the stimulus dimension of interest (e.g. brightness, pitch, etc.). On each trial the participant is asked to make a (typically dichotomous) perceptual judgment regarding the variable of interest. After numerous presentations of each level of the stimulus variable the experimenter can then plot perceptual performance as a function of the stimulus variable. By fitting a psychometric function to these data, the experimenter can infer not only the level of the stimulus variable at which perceptual performance transitions from one perceptual category to the other (the so-called Point of Subjective Equivalence (PSE)), but also the precision with which the participant is able to perceptually differentiate stimuli along this dimension (the Just Noticeable Difference (JND)) – with better precision indexed by steeper estimates of the fitted slope.

For the present study, the MOCS paradigm was applied to a Body Categorization Task in which the task-relevant stimulus dimension involved systematic variation in visual representations of male muscularity or body fat. This Body Categorization Task required participants to make dichotomous categorization decisions about sequentially presented images of male bodies. In one experimental block participants were instructed to categorize bodies varying in their level of muscularity as either ‘scrawny’ or ‘muscular’. In the other experimental block participants were asked to categorize body stimuli varying in their representation of body fat percentage as either ‘skinny’ or ‘fat’.

**Figure 1:**
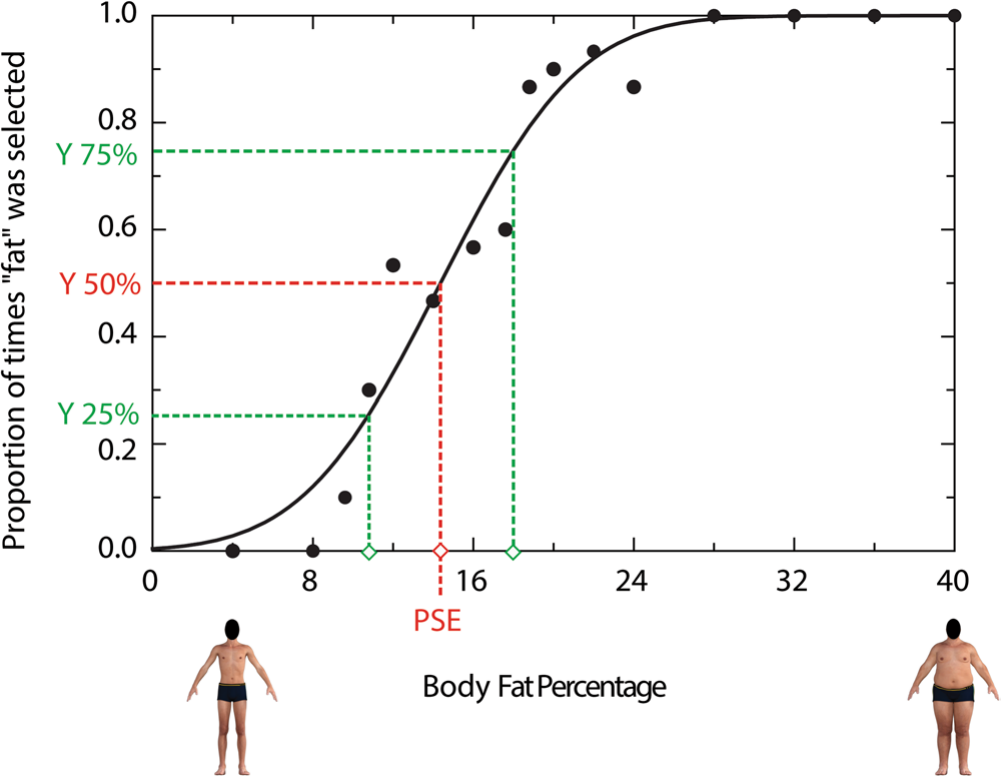
An example of how to calculate the body fat judgment PSE and JND for an individual participant’s body categorization data. The x-axis represents physical variation in the body fat dimension. The y-axis indicates the proportion of times each body was categorized as ‘fat’. The PSE was taken as the value on the x-axis that corresponds with 50% ‘muscular’/‘fat’ response rate. The JND was calculated by subtracting body values associated with 25% response rates from those yielding 75% response and dividing this value by two. Note: human figures displayed in this figure are computer generated.

Our Body Categorization Task provides three indices of psychophysical performance: PSE; JND and the average Response Time (RT) taken to make each categorization decision. The body stimuli value at a ‘muscular/fat’ response rate of 50% was taken as the PSE. JNDs – estimates of categorization precision – were obtained from these fits by subtracting x-axis body values associated with 25% response rates from those yielding 75% response and dividing this value by two [JND = ($\frac{y\,75 \% -y\,25 \% }{2}$)]. Figure 1 shows an example of how to calculate the body fat judgment PSE and JND for an individual participant’s body categorization data (body fat categorization, in this example).

**Figure 2:**
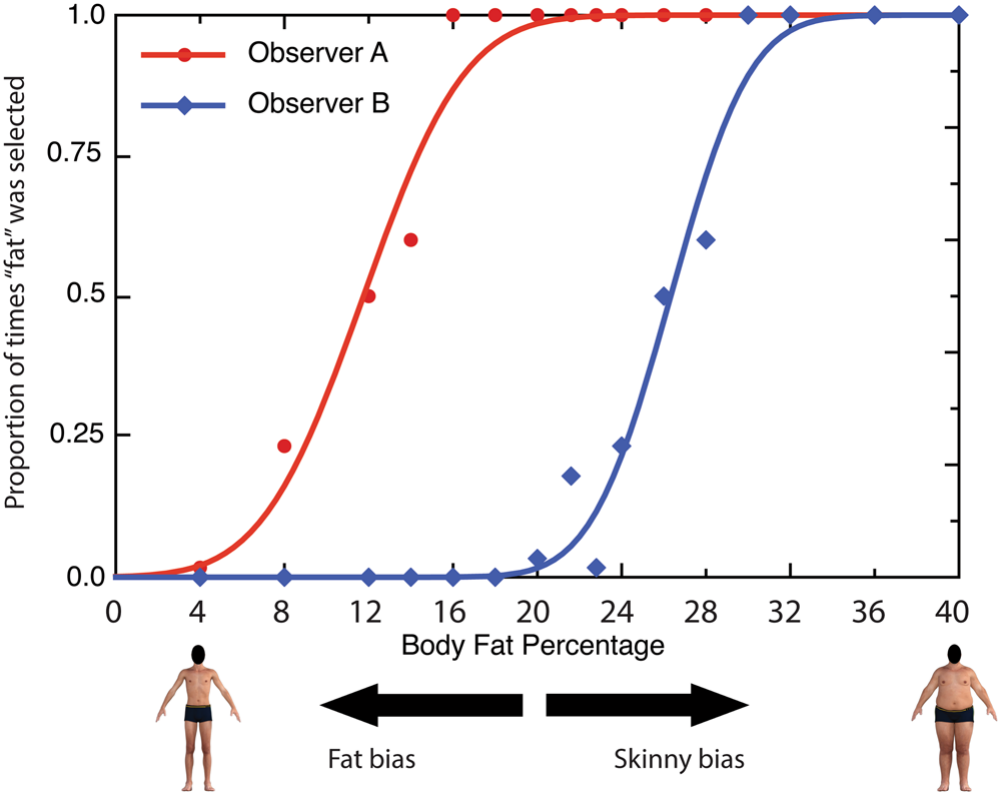
A demonstration of two individual PSE variations. The red curve implies a “fat” response bias and blue curve “skinny” bias a relatively high PSE. The x-axis depicts the degree of body fat percentage represented in the stimuli. The y-axis depicts the portion of times the participant categorized the presented body as ‘fat’. Note: human figures displayed in this figure are computer generated.

For the Body Categorization Task, the PSE indicates the level (i.e. magnitude) of the body variable at which an individual’s dichotomous report changes from ‘skinny’ to ‘fat’ in the body fat stimuli block, or from ‘scrawny’ to ‘muscular’ in the muscularity stimuli block. An example of such a transition from one perceptual categorization judgment to another can be seen in Fig. 2. Here the y-axis corresponds the proportion of trials in which a participant (or participants) classified the bodies they were shown as ‘fat’ rather than ‘skinny’. The x-axis represents systematic physical variation in the visual representation of body fat presented across trials. Consider the response distribution of two participants, *Participant A* and *Participant B*. *Participant A* might have a tendency to classify bodies as ‘fat’, compared to *Participant B*, who tends to make fewer ‘fat’ judgments across trials. For *Participant A*, this would manifest psychophysically as a shift to the left of the psychometric function (more ‘fat’ judgments), yielding a lower PSE estimate than *Participant B*, whose psychometric would be shifted to the right, signifying fewer ‘fat’ judgments.

**Figure 3:**
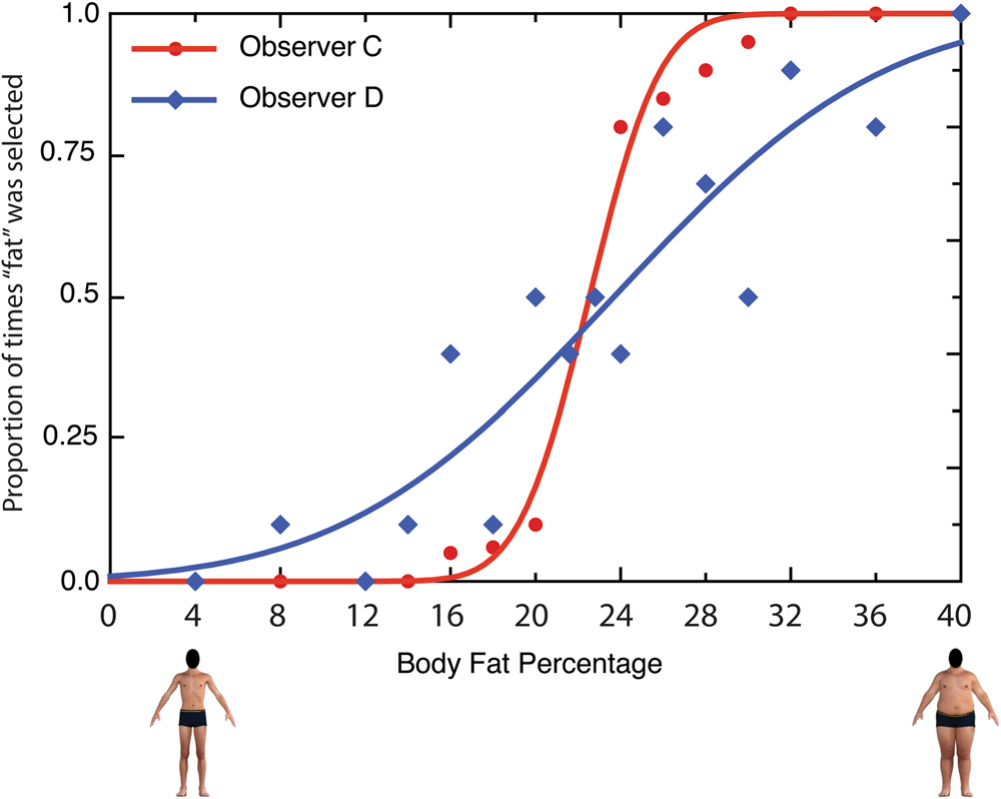
An illustration of response categorization data from two different participants, each exemplifying different levels of categorization precision: relatively high precision and low JND (red data and curve); and a relatively poor precision and high JND (blue data and curve). The x-axis depicts the degree of body fat percentage represented in the stimuli. The y-axis depicts the portion of times the participant categorized the presented body as ‘fat’. Note: human figures displayed in this figure are computer generated.

JND was taken as an index of the precision with which an individual shifted from one perceptual category to the other. Consider the response distribution of two additional participants, *C* and *D* (see Fig. 3). In the case of *Participant C*, their transition from one perceptual classification (‘skinny’) to the other (‘fat’) is more abrupt when expressed as a function of our physical manipulation of muscularity (x-axis) than *Participant D*, which is more gradual. This implies that there is greater uncertainty in *Participant D’s* transition from ‘skinny’ to ‘fat’ categorizations relative to *Participant C*, who requires smaller units of physical change in the fat dimension to achieve the same perceptual transition. That is to say, the shallower slope seen in *Participant D’s* data implies a larger JND (and therefore less precise categorization) than *Participant C*, whose categorization is more precise (smaller JNDs). Averaged RT refers to the average amount of time (in milliseconds) that a participant took to make each categorization judgment, from the time of body stimulus presentation to the time of the response button press.

Conceivably, JND and averaged RT could predict body dissatisfaction. Previous research has implicated the avoidance of body stimuli as a compensatory measure in those suffering from eating disorders^14^. It is theorized that avoidance behaviour could be used to escape from negative emotional experiences triggered by body stimuli^14,15^. For participants high in body dissatisfaction and eating disorder symptoms, this avoidance behavior might be reflected in the precision of categorization performance (as measured by JND). Avoidance would likely result in inconsistent processing of some of the presented stimulus (i.e. muscular and obese stimuli). As participants high in body dissatisfaction would likely avoid body stimuli^14^, it is likely that they would make categorization decisions based on less visual information compared to those who do not avoid stimuli, thus resulting in less precise categorizations for those high in body dissatisfaction (reflected by a greater JND). Avoidance behavior might also result in slower categorization decisions (greater RT) for those high in body dissatisfaction and eating disorder symptoms as initial avoidance of body stimuli may have to be overcome before the categorization decision can take place. Conversely, avoidance might motivate participants to respond faster in order to remove the body image from view. This could result in faster RTs and reduced accuracy on other indices.

Additionally, a prior study has demonstrated that females with anorexia nervosa vary in terms of their ability to detect changes in body size^16^. This study demonstrated within a group of individuals diagnosed with anorexia nervosa, those with lower weight were extremely sensitive to body size changes within the low BMI range whilst those registering heavier weight were less sensitive to body size changes. The same relationship could be reflected in the present study though relationships between JND and reported eating disorder symptoms.

Arguably, our Body Categorization Task offers certain advantages over traditional figural rating scales as a measure of body dissatisfaction. For instance, the use of multiple presentations of each body stimulus; random sequence of different levels of body stimuli; male body stimuli with variations in body fat and muscularity; and a greater number of body images (32 unique body images per body dimension) represented than the majority of existing figural rating scales.

The present study aims to explore the relationship between psychophysical performance indices derived from our Body Categorization Task (PSE, JND, and RT) and conventional psychological measures of male body image, such as body dissatisfaction (measured by the Male Body Attitude Scale; MBAS), eating disorder symptomology (measured by the Eating Disorders Examination Questionnaire; EDE-Q), and biometric data (Body Mass Index (BMI), body fat percentage, and Fat Free Mass Index (FFMI)). Given that these psychophysical measures might be a more objective tool with which to measure subjective categorization of physical dimensions (body fat and muscularity, in this case) this study aimed to compare PSE, JND, and averaged RT to a conventional figural rating scale (the Visual Body Scale for Men; VBSM)^17^ in terms of sensitivity for predicting body dissatisfaction.

## Method

### Participants

Eighty-two male undergraduate students from Western Sydney University (Age range = 17–35, *M* = 21.89, *SD* = 4.20) consented to participate in the study. An a-priori power analyses was conducted using G-POWER (alpha = 0.05, beta = 0.2) to inform an appropriate sample size for Spearman’s correlation analyses. This power analysis indicated that 82 observes were required for a medium effect size (ρ = 0.3) for a two-tailed test^18^. From the sample, 52% of participants identified as Caucasian, 12% of participants identified as North or South-East Asian, 16% of participants identified as Southern or Central Asian, 12% of participants identified as African or Middle Easter, and 8% of participants identified as other. Participants received course credit or a $20AUD gift voucher in compensation for their time. Ethical approval to conduct the present study was provided by the Western Sydney University Human Research Ethics Committee (ethics ID: H11778). This study is complied with APA ethical standards and guidelines.

### Measures

#### Body Categorization Task

**Figure 4:**
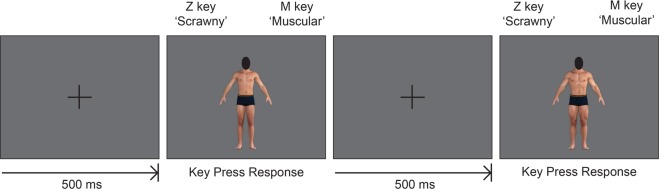
Example of Body Categorization Task trial sequence. Note: human figures displayed in this figure are computer generated.

The Body Categorization Task required participants to make a series of dichotomous categorization decisions about male bodies. Images of male bodies were presented on a computer screen one at a time. Depending on the trial, participants were required to label the body skinny or fat (body fat Body Categorization Task), or scrawny or muscular (muscular Body Categorization Task) via keypress response. After the keypress response was made, the presented body would disappear and be replaced by a new male body. Participants were then required to make another categorization decision. Figure 4 shows an example of the Body Categorization Task trial sequence.

The Body Categorization Task was comprised of 16 different levels of stimuli for skinny/fat judgements, and 16 different levels of stimuli for scrawny/muscular judgements. Twenty stimuli (10 body images for skinny/fat and scrawny/muscular judgments, respectively) used in the Body Categorization Task were directly adapted from the VBSM^17^ and the New Somatomorphic Matrix-Male (NSM-M)^19^. The bodies employed in these visual scales were perceptually representative of specific quantities of both body fat percentage and FFMI, as developed and validated using a series of psychophysical matching to existing validated images of male bodies^20^. The body stimuli that were most consistently matched to these validated images were taken as perceptually equivalent, and used to construct both figural rating scales. For example, the skinniest body adapted from the VBSM body fat scale accurately represents a male body with 4% body fat (body number 1 in the present study). The largest body adapted from the VBSM body fat scale represents a male body with 40% body fat (body number 16 in the present study). An additional 12 body stimuli (6 for skinny/fat judgements, 6 for muscularity judgements) were generated specifically for the present study using DAZ Studio 4.9. These additional bodies fell between body values presented in the VBSM and NSM-M.

All body numbers and associated body fat percentage and FFMI values used in the present study are shown in Table 1. Each body stimulus was presented 30 times, meaning that each participant made a total of 480 skinny/fat categorization judgments, and 480 scrawny/muscular judgements. The order of stimuli presentation was randomized.

**Table 1:** Body numbers and associated Body Fat Percentage and FFMI for Body Categorization Task Stimulus.

Body Number	1	2	3	4	5	6	7	8	9	10	11	12	13	14	15	16
Body Fat Percentage	4	8	12	14	16	18	20	21.32	22.64	24	26	28	30	32	36	40
FFMI (kg/m^2^)	16.5	18	19.5	19.8	21	21.75	22.5	23	23.49	24	24.75	25.5	26.25	27	28.5	30

#### Figural Rating Scale - Visual Body Scale for Men (VBSM)

**Figure 5:**
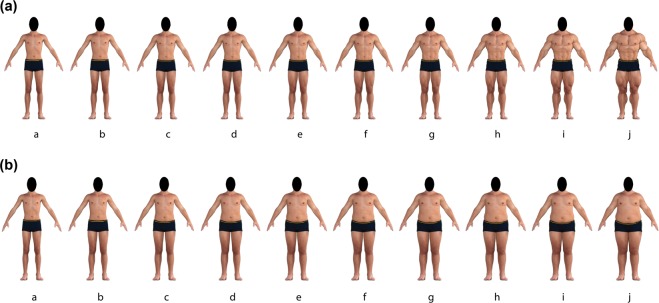
(**a**) The VBSM-M. Body stimuli ranges in FFMI from 16.5 kg/m^2^ (*body a*) to 30 kg/m^2^ (*body j*). (**b**) The VBSM-BF. Body stimuli ranges in body fat from 4% (*body a*) to 40% (*body j*). Note: human figures displayed in this figure are computer generated.

The VBSM^17^ is comprised of two figure rating scales: the Visual Body Scale for Men-Muscularity (VBSM-M); and the Visual Body Scale for Men-Body Fat (VBSM-BF). Figure rating scales are visual tools used to measure body image. Typically, figure rating scales present a number of images of bodies ordered in terms of increasing body fat or muscularity on a numbered scale. From the presented bodies, participants are asked to select the body that best approximates the visual appearance of their own body, and in separate trials, the body that best corresponds to how they would ideally wish their body to appear. The difference between the values of these two selected bodies is then used as an index of body dissatisfaction^1^. Additionally, participants were also asked to select which body best represents the average Australian male body. In this way, the VBSM was used to measure participants’ *perceived*, *average*, and *desired* body shape, respectively. Each of these three constructs was measured in terms of both body fat (VBSM-BF) and muscularity (VBSM-M), respectively. The VBSM-M consisted of 10 male bodies increasing in in Fat Free Mass Index (FFMI), ranging from 16.5–30 kg/m^2^ (Fig. 5a). The VBSM-BF consisted of 10 male bodies increasing in body fat percentage, ranging from 4–40% body fat (Fig. 5b). Figure rating scales were presented on a 15.4-inch (diagonal) LED monitor (screen resolution = 1280 × 800). Figure rating scales were presented with one trial consisting of 10 side-by-side male body images (either the VBSM-BF or VBSM-M) ordered in terms of increasing size. The VBSM-BF and -M appeared on the screen (total image size = 1034 × 166 px) and remained until a key-press response was made. The discrepancy between their selected ‘*perceived*’ and ‘*desired*’ body (calculated by subtracting ‘VBSM *perceived*’ scores from the ‘VBSM *desired*’ scores) was used as an index of body dissatisfaction. For example, if an participant were to select their *perceived* body fat as body g (28% body fat), and their *desired* body as body b (8% body fat), then their VBSM-BF dissatisfaction score would be 28–8 (body g – body b) = 20% body fat^17^.

For each body, participants were asked “*Is the presented body scrawny or muscular?*”. The bodies included for presentation were taken from a bank of 16 rendered male bodies (each body presented 30 times). Each of the 16 bodies varied in Fat Free Mass Index (FFMI; a measure of muscularity), ranging from 16.5–30 kg/m^2^ for the body muscle block. For the body fat block stimulus presentation, the body stimuli varied in body fat percentage (ranging from 4–40%) and participants were asked “*Is the presented body skinny or fat?*” Categorization choice and RT was recorded for each individual trial.

#### Male Body Attitudes Scale (MBAS)

The MBAS is a self-report questionnaire used to measure male body dissatisfaction^21^. The MBAS includes 24 items and assesses three dimensions of body attitude (muscularity, low body fat, and height), and has good internal reliability, test–retest reliability, and validity^21^. Items include “*I think I have too little muscle on my body*” (muscularity subscale), “*I think my body should be leaner*” (low body fat subscale), and “*I wish I were taller*” (height subscale)^21^. Each item on the MBAS uses a six-point scale that ranges from 0 (never) to 5 (always). In the present study, Cronbach’s *α* were 0.91 and 0.92 for the muscularity and body fat subscales, respectively.

#### Eating Disorder Examination Questionnaire (EDE-Q)

The EDE-Q is a self-report measure of eating disorder symptoms. The EDE-Q has been adapted from the Eating Disorder Examination interview^10^. The EDE-Q consists of 28 items, comprising four subscales: Restraint, Eating Concern, Shape Concern, and Weight Concern, and further provides a Global Score. Participants are required to rate the frequency or severity of core eating disorder symptoms, and related psychopathological behaviors (such as objective binge eating episode frequency) and beliefs over the past 28 days. Each item on the EDE-Q uses a seven-point Likert scale, with questions such as “*Have you had a strong desire to lose weight?*”, and “*How dissatisfied have you been with your weight?”*. The EDE-Q presents sufficient psychometric properties in female populations^22^, and moderate preliminary psychometric properties in male^23,24^. Of note, only EDE-Q subscale scores were used in the analysis. In the present study, Cronbach’s *α* were 0.76, 0.73, 0.87, 0.70, and 0.91 for Restraint, Eating Concern, Shape Concern, Weight Concern subscales, and Global EDE-Q score, respectively.

#### Biometric Data- Body mass index (BMI), body fat and fat free mass index (FFMI)

Date of birth and height (measured to 0.1 cm using a fixed Stadiometer) was obtained for each subject. These details were then entered into Healthy Edge V1.6.0, a software package that supports the Body Composition Monitor Scales. Body Mass Index (BMI) and body fat percentage were then obtained via Tanita BC-1000 Wireless Body Composition Monitor Scales^25^. Prior research has shown that Tanita Body Composition technology is accurate in providing measurements of body fat percentage and BMI, relative to traditional measures (e.g., measurements skinfold thickness^26^) and to dual-energy x-ray absorptiometry, the most accurate measure of body composition^27^. Fat Free Mass Index (FFMI; a biometric measure of a male’s degree of muscularity)^28^ and was also obtained. The following formula was used to calculate FFMI, with weight (kilograms) represented as W, body fat percentage represented as BF, and H is height (meters).: FFMI = W × [(100 − BF)/100] × H-2 + 6.1 × (1.8 − H).

### Procedure

Testing was conducted in a low illumination laboratory setting in order to minimize visual distraction. Testing consisted of one practice block and three test blocks. All tasks were presented on a 15.4-inch (diagonal) LED monitor (screen resolution = 1280 × 800). Viewing distance was fixed at 340 mm.

Participants completed three blocks of testing: (1) the muscular Body Categorization Task, (2) the body fat Body Categorization Task, and (3) self-report measures, including the MBAS, EDE-Q, and a basic six item demographic questionnaire, and the VBSM-M and VBSM-BF. The sequence of the three blocks was randomized between participants in order to account for order effects. Each figure presented in the Body Categorization Task was 166 pixels in height, and different levels of each body where presented randomly across trials in order to prevent order effects. Participants’ height was then measured using a fixed Stadiometer Finally, date of birth was obtained. These details were entered into Healthy Edge, a software package that provides a user interface for Body Composition Monitor Scales. Lastly, participants were instructed to stand on the Tanita BC-1000 Body Composition Monitor Scales without socks in order to record their weight, BMI, and body fat percentage.

### Statistical Analysis

#### Body Categorization Task

For each categorization task block (muscularity and body fat categorization; 480 trials per block) the proportion of ‘muscular’ or ‘fat’ responses were plotted as a function of the body stimulus value (Fat Free Mass Index (FFMI) or body fat percentage). A cumulative Gaussian curve was then fit to these data using a Levenburg-Marquardt algorithm maximum likelihood fitting procedure (see curve in Fig. 1). The lower and upper asymptotes (y-axis) were fixed at 0% and 100% respectively. The body stimuli value at a ‘muscular/fat’ response rate of 50% was taken as the PSE. JNDs – estimates of categorization precision – were obtained from these fits by subtracting x-axis body values associated with 25% response rates from those yielding 75% response and dividing this value by two [JND = ($\frac{y\,75 \% -y\,25 \% }{2}$)].

#### Additional Analyses

All six Body Categorization Task values, including PSE, JND, and average RT (for both muscular and body fat categorization judgments) were each subjected to Spearman’s correlation analysis with both MBAS subscales (muscle dissatisfaction and body dissatisfaction), all four EDE-Q subscales, and participants’ BMI, FFMI, and body fat percentage. Additionally, the VBSM-M and VBSM-BF (*average*, *perceived*, *desired*, and dissatisfaction scores) were each correlated with both MBAS subscales all four EDE-Q subscales, and participants’ BMI, FFMI, and body fat percentage. Finally, the Body Categorization Task PSE, JND, and average RT (for both muscular and body fat categorization judgments) were each correlated with VBSM-M and VBSM-BF *average*, *perceived*, *desired*, and dissatisfaction scores. In order to control for type-1 error, the Benjamini-Hochberg method (False Discovery Rate control) was utilized^29^.

Based on inspection of scatterplots assumptions for linearity and homoscedasticity were met for each correlational analysis. Additionally, standardized residuals were examined for multivariate outliers. This analysis returned no multivariate outliers.

## Results

The data from one participant were excluded from subsequent analysis due to poor perceptual categorization fits. Table 2 displays descriptive information for our sample.

**Table 2:** Means, Standard Deviation, and Range of participants’ age, MBAS scores, EDE-Q scores, and Physiological Data.

	*M*	*SD*	Range
Age	21.89	4.20	17–35
MBAS			
Global	72.54	19.96	30–131
Muscularity Subscale	43.18	13.42	17–82
Body Fat Subscale	36.04	12.98	14–71
EDE-Q			
Restraint	1.11	1.28	0.0–5.4
Eating Concern	0.51	0.61	0.0–3.0
Shape Concern	1.89	1.43	0.0–5.4
Weight Concern	1.48	1.18	0.0–4.6
Physiological Data			
BMI	25.39	5.52	17.0–43.4
Body Fat Percentage	18.59	8.02	6–41
FFMI	20.30	2.38	16.09

*Note*. MBAS = Male Body Attitude Scale; EDE-Q = Eating Disorder Examination Questionnaire; BMI = Body Mass Index; FFMI = Fat Free Mass Index.

Spearman’s correlations between muscular Body Categorization Task psychophysical indices, and figural rating scale values (*average*, *perceived*, *desired*, and dissatisfaction scores), and MBAS, EDE-Q, and physiological variables for the remaining participants are shown in Table 3. Results showed that muscular JND was significantly positively correlated with Weight Concern. Additionally, VBSM-M *perceived* scores were positively correlated with all three biometric measures, and VBSM-M dissatisfaction scores were positively correlated with MBAS Muscularity Dissatisfaction, and negatively correlated with all three biometric measures.

**Table 3:** Spearman’s correlations between PSE, JND, RT, VBSM scores for ‘scrawny/muscular’ categorizations, and MBAS scores EDE-Q scores, and physiological variables.

Measure	MBAS			EDE-Q			Biometric Variables		
	Muscularity	Body Fat	Restraint	Eating Concern	Shape Concern	Weight concern	BMI	Body Fat Percentage	FFMI
PSE	−0.037	−0.043	0.050	−0.135	−0.033	0.007	0.008	−0.036	0.046
JND	0.134	0.097	0.085	0.190	0.208	0.257*	−0.093	−0.69	−0.85
RT	−0.080	0.044	0.077	−0.081	0.002	0.015	0.137	0.142	0.115
VBSM-M									
Average	−0.064	−0.154	−0.198	−0.134	−0.187	−0.060	−0.108	−0.104	−0.118
Perceived	−0.306**	0.053	0.212	0.033	−0.014	0.107	0.420***	0.355**	0.450***
Desired	−0.008	−0.012	0.093	0.057	0.042	0.142	0.147	0.067	0.219*
Dissatisfaction	0.279*	−0.095	−0.177	0.001	0.020	−0.030	−0.292**	−0.268*	−0.289**

*Note*. ***p < 0.001, **p < 0.01, *p < 0.05; PSE = Point of Subjective Equivalence; JND = Just Noticeable Difference; RT = Average Response Time; VBSM-M = Visual Body Scale for Men-Muscularity; *Perceived* = selected ‘*perceived*’ body; Desired = selected ‘*desired*’ body; Dissatisfaction = selected ‘*perceived*’ body minus selected; MBAS = Male Body Attitude Scale; EDE-Q = Eating Disorder Examination Questionnaire; BMI = Body Mass Index; FFMI = Fat Free Mass Index.

Spearman’s correlations between body fat Body Categorization Task psychophysical indices, and figural rating scale values (*average*, *perceived*, *desired*, and dissatisfaction scores), and MBAS, EDE-Q, and physiological variables are shown in Table 4. Results showed that body fat RT was positively correlated with MBAS body fat dissatisfaction, Eating Concern, Weight Concern, and Shape Concern. Additionally, the VBSM-M *perceived* scores and dissatisfaction scores were both positively correlated with MBAS body fat dissatisfaction scores, all four subscales of the EDE-Q, and all three biometric measures.

**Table 4:** Spearman’s correlations between PSE, JND, RT, VBSM scores for ‘skinny/fat’ categorizations, and MBAS scores EDE-Q scores, and physiological variables.

Measure	MBAS			EDE-Q			Biometric Variables		
	Muscularity	Body Fat	Restraint	Eat Concern	Shape Concern	Weight concern	BMI	Body Fat Percentage	FFMI
PSE	−0.127	−0.081	0.081	−0.159	−0.082	−0.049	0.060	0.064	0.066
JND	0.061	0.034	0.018	0.137	0.072	−0.016	0.051	0.107	0.033
RT	0.183	0.233*	0.218	0.253*	0.229*	0.259*	0.162	0.165	0.135
VBSM-BF									
Average	−0.060	−0.024	0.023	0.041	0.007	0.024	0.168	0.170	0.133
Perceived	0.001	0.602***	0.538***	0.365***	0.475***	0.498***	0.825***	0.816***	0.772***
Desired	−0.052	−0.062	−0.063	−0.233*	−0.101	0.090	0.177	0.153	0.188
Dissatisfaction	0.063	0.686***	0.628***	0.541***	0.597***	0.510***	0.737***	0.745***	0.677***

*Note*. ***p < 0.001, **p < 0.01, *p < 0.05; PSE = Point of Subjective Equivalence; JND = Just Noticeable Difference; RT = Average Response Time; VBSM-BF = Visual Body Scale for Men-Body Fat; *Perceived* = selected ‘*perceived*’ body; Desired = selected ‘*desired*’ body; Dissatisfaction = selected ‘*perceived*’ body minus selected; MBAS = Male Body Attitude Scale; EDE-Q = Eating Disorder Examination Questionnaire; BMI = Body Mass Index; FFMI = Fat Free Mass Index.

Spearman’s correlations between PSEs, JNDs, and RTs obtained for muscularity and body fat categorizations and corresponding figural rating scale judgments are shown in Table 5. For both muscularity and body fat judgements, PSE positively correlated with VBSM *average* scores, and muscular PSE positively correlated with VBSM-M *perceived* and *desired* scores. Spearman’s correlations between PSE, JNDs, and RTs for both muscularity and body fat categorizations are shown in Table 6. Results showed that muscularity PSEs, JNDs, and RTs were each positively correlated with their equivalent body fat categorization measurement. Additionally, PSE and RT were both negatively correlated for both categorization types.

**Table 5:** Correlations between VBSM scores and PSE, JND, and RT for both Muscularity and Body Fat Categorization Judgments.

	Muscularity			Body Fat		
	PSE	JND	RT	PSE	JND	RT
VBSM-M						
Perceived	0.26*	−0.01	0.06	0.05	0.09	0.01
Desired	0.31**	0.20	−0.22	0.05	0.18	−0.13
Dissatisfaction	−0.04	0.14	−0.14	−0.03	0.03	−0.03
Average	0.50***	0.28*	−0.22	0.04	0.21	−0.16
VBSM-BF						
Perceived	0.13	0.08	0.01	0.15	0.14	0.10
Desired	0.19	−0.01	−0.07	0.19	0.07	−0.20
Dissatisfaction	0.04	0.10	0.03	−0.03	0.11	0.21
Average	0.18	−0.03	0.05	0.35**	0.21	−0.03

*Note*. ***p < 0.001, **p < 0.01, *p < 0.05; VBSM-M = Visual Body Scale for Men-Muscularity; VBSM-BF = Visual Body Scale for Men-Body Fat; *Perceived* = selected ‘*perceived*’ body; Desired = selected ‘*desired*’ body; Dissatisfaction = selected ‘*perceived*’ body minus selected ‘*desired*’ body; Average = selected ‘average’ body; PSE = Point of Subjective Equivalence; JND = Just Noticeable Difference; RT = Response Time.

**Table 6:** Correlations between PSE, JND, and RT for both Muscularity and Body Fat Categorization Judgments.

	Muscularity			Body Fat	
	PSE	JND	RT	PSE	JND
Muscularity					
PSE	—	—	—	—	—
JND	0.06		—	—	—
RT	−0.32**	−0.26*		—	—
Body Fat					
PSE	0.35**	−0.08	−0.21	—	—
JND	0.28**	0.41***	−0.20	0.20	—
RT	−0.31**	−0.02	0.60***	−0.40***	−0.26*

*Note*. ***p < 0.001, **p < 0.01, *p < 0.05; PSE = Point of Subjective Equivalence; JND = Just Noticeable Difference; RT = Response Time.

## Discussion

The present study aimed to examine the relationship between our three behavioral indices of perceptual categorization performance (PSE, JND, and average RT) and conventional physiological and psychological correlates of male body image and body dissatisfaction. Additionally, this study aimed to compare the predictive utility of these measures of perceptual categorization performance to the more standard Visual Body Scale for Men (VBSM)^17^.

We find that our participants’ body fat and the muscularity categorization PSEs are not significantly associated with either body dissatisfaction (MBAS scores) or eating disorder symptoms (EDE-Q scores). That is to say, the point at which judgments shift from one perceptual body category to another (i.e. skinny to fat, scrawny to muscular) is not associated with eating behavior or attitudes toward one’s own body.

When examining correlations between PSE and the VBSM, we found no significant correlations between body fat PSEs and *perceived* or *desired* Visual Body Scale for Men – Body Fat (VBSM-BF) ratings. *Average* VBSM-BF ratings were, however, positively correlated with body fat PSEs. With regards to judgments of muscularity, *perceived* and *desired* Visual Body Scale for Men – Muscularity (VBSM-M) ratings were both positively correlated with muscularity PSEs. That said, an even stronger positive correlation was observed between muscularity PSE and VBSM-M *average* ratings. Taken together these results suggest that participants may have relied more heavily on their internal representation of what constitutes the average male body (in terms of body fat and muscularity) to inform their categorization judgments than either their *perceived* or *desired* body ratings. No other correlations were found with the PSE.

JNDs for muscularity categorization judgments were positively correlated with the Weight Concern subscale from the EDE-Q, meaning that participants with greater concern about their weight were less precise in their perceptual categorization of body muscularity. There are several possibilities that could explain these results. It could be that weight satisfied individuals might themselves possess more muscular bodies which provide them better visual access to muscle groups than those who are weight dissatisfied. However, this explanation is not supported by the data as no significant association between muscular JND and FFMI was found. An alternative explanation could lie in central coherence differences between participants with high and low eating disorder symptomatology. A series of clinical studies have shown that individuals suffering from eating disorders display weak central coherence compared to healthy controls^30^. Behaviorally, this has been found to manifest as a tendency to focus on specific details at the expense of configural information^31^. This could be meaningful for our task as there is a distinct difference in visual complexity between muscular and obese stimuli used in the Body Categorization Task. The muscular stimuli used in our scrawny/muscular categorization task possess a multitude of features (the shape and tone of various muscle groups comprising the muscular male torso). These same features are not present in the bodies used in our skinny/fat categorization task. When assessing muscular bodies, those high in eating disorder symptomatology may conceivably focus their attention on different local bodily features from trial to trial. For example, in *trial 1* they may focus primarily on pectoral muscles and upper abdominal muscles, whereas in *trial 2* they may focus on areas of biceps and quadriceps muscle groups. If categorization decisions were made based on a different combination of features across trials, then categorizations might be less precise (higher JNDs). Conversely, bodies presented in our skinny/fat categorizations possess comparatively fewer diagnostic features, and therefore afford less opportunity for featural processing variance across skinny/fat trials, and hence better categorization precision irrespective of eating disorder symptomology. Alternatively, those who are more satisfied may simply adopt more efficient categorization strategies – which may involve perceptual analysis of global or local features. Which of these alternatives, singularly or in combination, explain our observed relationship between JND and eating disorder symptoms is difficult to say based the evidence presented here. Future studies employing eye-tracking in conjunction with our categorization task may shed light on these various possibilities. No other correlations were found with the JND.

Response time (RT) analyses indicate a positive relationship between the time taken to make skinny/fat categorizations, and body fat dissatisfaction (MBAS) and three of the subscales of the EDE-Q (Eating Concern, Shape Concern, and Weight Concern). This might reflect either hypervigilance and rumination, or avoidance behavior for those high in body dissatisfaction and eating disorders^14^. According to this interpretation, when body dissatisfied individuals make fat-related body categorizations, they first pay careful attention to the details and then some avoid^32^. Consequently, their decisions may be delayed until their hypervigilance or avoidance is overcome. Over the course of the experimental block of trials this would manifest as longer RTs for participants with high body dissatisfaction relative to those with low dissatisfaction. Curiously, an analogous result was not found for scrawny/muscular categorizations as RTs did not significantly correlate with any body image-related psychological measure. This apparent inconsistency may reflect the male body types (i.e. ideal and non-ideal) present in each categorization task. Skinny/fat categorizations involve categorizing along a dimension (body fat) that is generally considered undesirable within the western cultural context. Typically, these culturally ‘non-ideal’ bodies are the body types implicated in avoidance cognitive models of eating disorders^14^. Conversely, scrawny/muscular categorizations involve stimulus variation along a body dimension that male respondents are more likely to consider ideal.

All three psychophysical indices of performance failed to significantly correlate with participants’ Body Mass Index (BMI), body fat percentage, or Fat Free Mass Index (FFMI) for both muscularity and body fat categorization tasks. It seems, therefore, that for the perceptual categorization tasks used in present study, participants were unlikely to have used their own level of body fat or level of muscularity as a reference against which to make their categorization decisions.

Results showed that muscularity PSEs, JNDs, and RTs were each positively correlated with their equivalent body fat categorization measurement. For PSEs this implies that individuals who categorized more bodies as “muscular” also categorized more bodies as “fat”. This might suggest that when making categorization judgments some individuals possess a general size bias, as opposed to specific muscle or fat biases. Positive correlations between muscular JND and body fat JND, and muscular RT and body fat RT suggest that precision and speed were consistent across blocks. This likely reflects that participants were inherently stable in their ability to make body categorization judgments, regardless of the specific categorization dimension. No other correlations were found to be significant.

In comparing the Body Categorization Task to the VBSM, we find that the VBSM was both highly and consistently correlated with body dissatisfaction (MBAS) and eating disorder symptoms (EDE-Q subscales), whereas the Body Categorization Task was not. VBSM-M *perceived* body scores significantly negatively correlated with the MBAS muscularity subscale, and positively correlated with BMI, body fat percentage and FFMI, demonstrating that those with lower perceived muscularity tend to be more dissatisfied with their own body’s muscularity. This implies that individuals are able to use the VBSM-M to accurately identify the muscular composition of their own body as those with higher FFMI and BMI selected *perceived* bodies that were greater in muscularity compared to those with lower FFMI and BMI. It should be noted that as BMI is calculated using unspecified mass, it can be highly affected by muscle mass, particularly in males^33^.

VBSM-M dissatisfaction scores also significantly positively correlated with the MBAS muscularity dissatisfaction subscale and negatively correlated with BMI, body fat percentage and FFMI. Simply put, participants with a greater discrepancy between their reported *perceived* and *desired* muscularity tended to be more dissatisfied with their muscles, and smaller (in both muscularity and body fat) in terms of their own body size. Notably, the VBSM-M did not correlate with any of the EDE-Q subscales. This is of interest because in this instance, muscularity JND showed to be a better predictor of eating disorder symptoms than the VBSM, returning significant positive correlations (see above).

VBSM-BF *perceived* body scores returned significant positive correlations with the MBAS body fat dissatisfaction subscale, all four subscales of the EDE-Q, BMI, body fat percentage, and FFMI. These results indicate that participants with higher *perceived* body fat (as indicated by their VBSM-BF score) were higher in BMI, body fat percentage, and FFMI. These participants were also more dissatisfied with body fat percentage and tended to have more pronounced eating disorder symptoms. Thus, the VBSM-BF is a good predictor of *perceived* body fat, consistent with previous research indicating associations between higher body fat percentage and BMI, and greater body dissatisfaction^4,5,34^ and eating disorder symptoms^35^.

VBSM-BF dissatisfaction scores were also all significantly positively correlated with the MBAS body fat dissatisfaction subscale, all four subscales of the EDE-Q, and BMI, body fat percentage, and FFMI. This suggests that the VBSM-BF dissatisfaction measure is sensitive in detecting body dissatisfaction and eating disorder symptoms, and analogous to the VBSM-BF *perceived* measure, demonstrates expected positive associations between BMI and body fat percentage, and body dissatisfaction^4,5,34^, and eating disorder symptoms^35^. Of note, VBSM-M and -BF *average* body scores were not significantly correlated with any body image-related psychological or physiological measures.

The present study shows that the PSE provided by our perceptual Body Categorization Task is not as sensitive in predicting self-report body dissatisfaction or eating disorder symptoms as the VBSM figural rating scale was. In relation to the PSE, a potential problem with the current task is that participants were not prompted to reference their own body when making each categorization judgment. This appears to be reflected in the finding that VBSM *average* body scores were the only consistent significant predictor of participants’ PSE for both body fat and muscularity. Future research should seek to utilize the MOCS paradigm employed in this study, but directly prompt participants to self-reference their own body, and hence associated attitudes/emotions. This could be achieved by instructing participants to indicate if the presented stimuli are smaller or larger than their own body, or smaller or larger than their desired body.

However, the psychophysical indices provided through the use of the MOCS showed potential as a predictor of body dissatisfaction. JND and average RT proved to be sensitive predictors of body dissatisfaction and eating disorder symptoms across participants. Of interest, there was a difference between JND and RT depending on the degree of eating disorder symptomology and body dissatisfaction, and the nature of the categorization judgment (skinny/fat or scrawny/muscular). Additionally, muscularity JND mapped onto participants’ weight concern, whilst the VBSM-M did not. JND was found to be a better indicator of eating disorder symptoms compared to the VBSM-M. This is a meaningful result as muscularity, and the way it relates to eating-related cognitions and behavior, is an essential factor in male body image^1,28,36^. Future research should aim to replicate this relationship with a larger sample, and employ eye-tracking. Additionally, future research should seek to examine the relationship between muscular categorization JNDs and eating disorder symptoms in a clinical male population (e.g., muscle dysmorphic and/or steroid users). If this relationship shows to be robust then the Body Categorization Task provide a more objective indicator of male eating disorder symptoms compared to existing self-report measures.

The Body Categorization Task holds the potential to remedy some existing problems with figural rating scales and provide a more objective measure of body dissatisfaction. Additionally, the Body Categorization Task, specifically the muscularity JND, offers additional predictive utility for eating disorder symptoms. RT also showed to be effective in predicting eating disorder symptoms and body dissatisfaction for skinny/fat categorizations. Although the results of the Body Categorization Task provide new information about how males with different levels of body dissatisfaction and eating disorder symptoms process and respond to visual body images, on balance the greater efficiency of the more conventional Rating Scale suggests may be more appropriate in clinical settings. That said, our muscular Body Categorization Task (JND) predicted an aspect of body shape concern not captured by the Figure Rating Scale. This implies that our body categorisation may prove to be a useful clinical research tool in research settings, potentially allowing for examination of cognitive styles and strategies (such as avoidance or hypervigilance) which may underlie body dissatisfaction and eating disorders.

## Supplementary information

Supplementary Material Scatter Plots
