# Supplementary material for: The relationship between psychophysical body categorization performance and male body dissatisfaction
Source: Sci Rep. 2019 Mar 7;9:3882. doi: 10.1038/s41598-019-40502-z (PMC6405907; doi:10.1038/s41598-019-40502-z)
Supplement: Supplementary file 1 — Supplementary Material Scatter Plots [file 41598_2019_40502_MOESM1_ESM.docx]

The relationship between psychophysical body categorization performance

and male body dissatisfaction

**Supplementary Material**

**Daniel Talbot, Evelyn Smith, and John Cass**

School of Social Sciences and Psychology, Western Sydney University, Bullecourt Ave, Milperra, Australia. 2214

**Corresponding author:**

Daniel Talbot

Email: d.talbot@westernsydney.edu.au

Phone: +61 422857844

|  |
| --- |
| *Figure 1.* Correlation between Muscular JND and EDE-Q Weight Concern. |

|  |
| --- |
| *Figure 2.* Correlation between Body Fat RT and MBAS Body Dissatisfaction. |

|  |
| --- |
| *Figure 3.* Correlation between Body Fat RT and EDE-Q Eating Concern. |

|  |
| --- |
| *Figure 4.* Correlation between Body Fat RT and EDE-Q Shape Concern. |

|  |
| --- |
| *Figure 5.* Correlation between Body Fat RT and EDE-Q Weight Concern. |

|  |
| --- |
| *Figure 6.* Correlation between PSE Muscularity and VBSM Perceived Muscularity. |

|  |
| --- |
| *Figure 7.* Correlation between PSE Muscularity and VBSM Desired Muscularity. |

|  |
| --- |
| *Figure 8.* Correlation between PSE Muscularity and VBSM Average Muscularity. |

|  |
| --- |
| *Figure 9.* Correlation between JND Muscularity and VBSM Average Muscularity. |

|  |
| --- |
| *Figure 10.* Correlation between PSE Body Fat and VBSM Average Body Fat. |
